# NitroSynapsin therapy for a mouse MEF2C haploinsufficiency model of human autism

**DOI:** 10.1038/s41467-017-01563-8

**Published:** 2017-11-14

**Authors:** Shichun Tu, Mohd Waseem Akhtar, Rosa Maria Escorihuela, Alejandro Amador-Arjona, Vivek Swarup, James Parker, Jeffrey D. Zaremba, Timothy Holland, Neha Bansal, Daniel R. Holohan, Kevin Lopez, Scott D. Ryan, Shing Fai Chan, Li Yan, Xiaofei Zhang, Xiayu Huang, Abdullah Sultan, Scott R. McKercher, Rajesh Ambasudhan, Huaxi Xu, Yuqiang Wang, Daniel H. Geschwind, Amanda J. Roberts, Alexey V. Terskikh, Robert A. Rissman, Eliezer Masliah, Stuart A. Lipton, Nobuki Nakanishi

**Affiliations:** 1 Neurodegenerative Disease Center, Scintillon Institute, San Diego, CA 92121 USA; 2 Neuroscience and Aging Research Center, Sanford Burnham Prebys Medical Discovery Institute, La Jolla, CA 92037 USA; 3 Center for Autism Research and Treatment (CART), University of California, Los Angeles, CA 90095 USA; 4 Bioinformatics Core Facility, Sanford Burnham Prebys Medical Discovery Institute, La Jolla, CA 92037 USA; 5 Department of Molecular Medicine and Neuroscience, Neuroscience Translational Center, The Scripps Research Institute, La Jolla, CA 92037 USA; 6 Institute of New Drug Research, Jinan University College of Pharmacy, Guangzhou, 510632 China; 7 Department of Neuroscience and Mouse Behavioral Assessment Core, The Scripps Research Institute, La Jolla, CA 92037 USA; 8 Department of Neurosciences, University of California, San Diego, School of Medicine, La Jolla, CA 92093 USA; 9 Veterans Affairs San Diego Healthcare System, San Diego, CA 92161 USA; 10 Present Address: Departament de PsiquiatriaiMedicina Legal, Institut de Neurociències, Universitat Autònoma de Barcelona, Bellaterra, Spain; 11 Present Address: Department of Molecular and Cellular Biology, University of Guelph, Guelph, ON Canada N1G 2W1; 12 Present Address: National Institute on Aging, NIH, Bethesda, MD 20892 USA

## Abstract

Transcription factor MEF2C regulates multiple genes linked to autism spectrum disorder (ASD), and human MEF2C haploinsufficiency results in ASD, intellectual disability, and epilepsy. However, molecular mechanisms underlying *MEF2C* haploinsufficiency syndrome remain poorly understood. Here we report that *Mef2c* ^+/−^(*Mef2c*-het) mice exhibit behavioral deficits resembling those of human patients. Gene expression analyses on brains from these mice show changes in genes associated with neurogenesis, synapse formation, and neuronal cell death. Accordingly, *Mef2c-*het mice exhibit decreased neurogenesis, enhanced neuronal apoptosis, and an increased ratio of excitatory to inhibitory (E/I) neurotransmission. Importantly, neurobehavioral deficits, E/I imbalance, and histological damage are all ameliorated by treatment with NitroSynapsin, a new dual-action compound related to the FDA-approved drug memantine, representing an uncompetitive/fast off-rate antagonist of NMDA-type glutamate receptors. These results suggest that *MEF2C* haploinsufficiency leads to abnormal brain development, E/I imbalance, and neurobehavioral dysfunction, which may be mitigated by pharmacological intervention.

## Abstract (web summary)

Human MEF2C haploinsufficiency results in Autism Spectrum Disorder (ASD), but it is unclear if the same is true in mice. Here, the authors show that *Mef2c* ^*+/−*^mice have behavioral defects and neuronal abnormalities similar to ASD, and symptoms can be ameliorated with the new drug, NitroSynapsin.

## Introduction

Myocyte enhancer factor 2 (MEF2) transcription factors belong to the MADS (MCM1-agamous-deficiens-serum response factor) gene family^1,2^. In brain, MEF2C is critical for neuronal differentiation, synaptic formation, and neuronal survival^3–7^. Recent gene expression analyses and genetic linkage experiments identified MEF2C as a convergent point in multiple regulatory pathways involved in the pathogenesis of autism spectrum disorder (ASD)^8,9^. We previously showed that conditional knockout of *Mef2c* in nestin-expressing neural progenitor cells produced mice with impaired electrophysiological network properties and behavioral deficits reminiscent of Rett syndrome, a neurological disorder related to autism spectrum disorder (ASD)^10^. Subsequent to our study, human geneticists mapped the overlapping regions of chromosome 5q14.3q15 microdeletions that cause neurological deficits in children, and identified *MEF2C* haploinsufficiency as the cause. These patients exhibit signs and symptoms that include ASD, intellectual disability (ID), poor reciprocal behavior, lack of speech, stereotyped and repetitive behavior, and epilepsy^11–22^. The disorders caused by *MEF2C* haploinsufficiency have been collectively termed *MEF2C* haploinsufficiency syndrome (MCHS)^22^. In addition, multiple MEF2 target genes have been identified as autism-related genes in human pedigrees with shared ancestry^23^.

One emerging mechanism for ASD pathogenesis is excitation/inhibition (E/I) imbalance in synaptic transmission, which may occur via several molecular pathways^24–27^. In *MeCP2*-deficient mice, a model of human Rett syndrome, impaired synaptic function and E/I imbalance lead to hyperexcitability in the hippocampus^28^. Furthermore, MeCP2 has been shown to influence the expression of MEF2C^29^. Interestingly, the *N*-methyl-d-aspartate-type glutamate receptor (NMDAR) antagonist memantine has been reported to improve hippocampal E/I imbalance in experimental models^30^. Memantine is an uncompetitive/fast off-rate NMDAR antagonist, which predominantly inhibits extrasynaptic receptors^31^; it has been approved by the US Food and Drug Administration (FDA) and European Medicines Administration (EMA) for moderate-to-severe Alzheimer’s disease^31^. However, to date, despite initial enthusiasm^32^, memantine has not been found to be effective for ASD in advanced human clinical trials, and in fact, at least one of these trials has been terminated due to lack of efficacy^33^. Thus, it appears that improved drugs may be needed if NMDAR antagonism is going to prove worthwhile for the treatment of ASD and related conditions. Along these lines, we recently synthesized an improved series of drugs based on dual memantine-like action and redox-based inhibition of extrasynaptic NMDARs; initially, these compounds were called “NitroMemantines,” but recently the lead compound, YQW-036/NMI-6979, was designated NitroSynapsin because of its ability to restore synaptic number and function in the face of multiple insults^34–36^.

In the present study, we develop *Mef2c* ^+/−^ (*Mef2c*-het) mice as a model for the human MEF2C haploinsufficiency form of ASD. We show that *Mef2c*-het mice display neuronal and synaptic abnormalities, decreased inhibitory and increased excitatory synaptic transmission in the hippocampus, suppressed long-term potentiation (LTP), and MCHS-like behavioral phenotypes. Importantly, we found that nearly all of these phenotypes are rescued or mitigated by chronic treatment with NitroSynapsin. This study therefore suggests that MEF2C haploinsufficiency induces neuronal and synaptic abnormalities that play an important role in ASD/MCHS-like behavioral phenotypes, which can be improved with pharmacological treatment.

## Results

### *Mef2c*-hets display autistic behavior and reduced viability

**Fig. 1:**
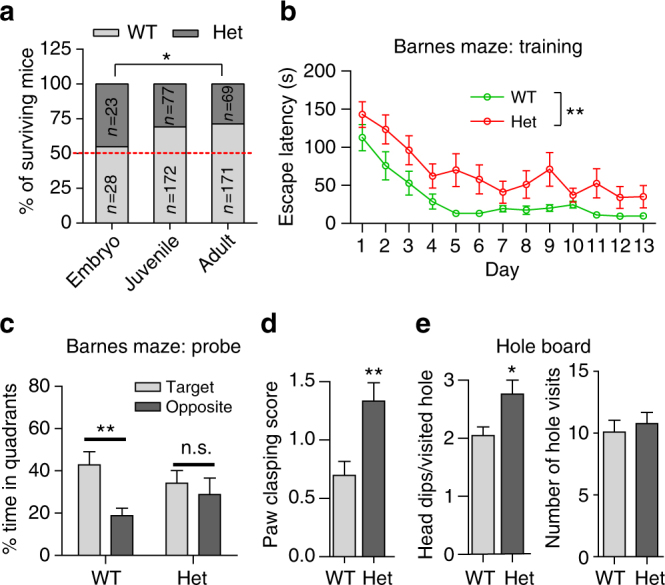
*Mef2c*-het mice display MCHS-like phenotypes. **a** *Mef2c*-het mice die prematurely. The number of *Mef2c*-het compared to WT mice was nearly equal at E18, but ~45% that of WT by adulthood (~3 months) (*P < 0.05 by *χ* ^2^). **b**, **c** Impaired spatial learned and memory in the Barnes maze of *Mef2c*-het mice during training (**b**) and on subsequent probe tests (**c**). **d**, **e** Increased paw clasping (**d**) and repetitive head dipping (**e**) of *Mef2c*-het mice in hole-board exploration. Data are mean ± s.e.m.; *n* = 9–11 mice per genotype in **b**, **c**, and **e**; *n* = 30 (WT) and 21 (het) in **d**; *P < 0.05, **P < 0.01 by Student’s *t* test (**c**–**e**) or ANOVA (**b**). n.s. not significant

MEF2C protein expression is significantly lower (*P* < 0.01) in *Mef2c*-het mice than in wild-type (WT) littermates (Supplementary Fig. 1), and we observed a significant number of early deaths in the*Mef2c*-het mice (Fig. 1a).We counted the number of viable animals from crosses between WT and *Mef2c*-het parents. While the number of WT and *Mef2c*-het offspring were approximately equal on embryonic day (E)18 (28 vs. 23, respectively), the ratio of surviving *Mef2c*-het to WT mice was 44% and 40% by postnatal day (P)21 and 90, respectively. The difference between survival at E18 and adult was significant (*P* *<* 0.05 by *χ* ^2^). In addition to reduced viability, *Mef2c*-het mice that survived to 3 months of age exhibited a decrease (~14%) in body weight compared to their WT counterparts (31.9 ± 1.0 g for WT vs. 27.4 ± 0.8 g for *Mef2c*-het; *P* < 0.001 by Student’s *t* test).

To determine whether adult *Mef2c*-het mice display MCHS-like phenotypes, we performed behavioral tests on male *Mef2c*-het mice and their WT littermates (≥3 months of age). Similar to human patients showing cognitive impairment, *Mef2c*-het mice performed poorly in the Barnes maze, a test that measures spatial learning and memory function. *Mef2c*-het mice took a significantly longer time to find the escape tunnel during training sessions (Fig. 1b). In subsequent probe tests,WT mice, but not *Mef2c*-het mice, showed a preference for the target quadrant compared to the opposite quadrant (Fig. 1c), suggesting impaired spatial memory in the *Mef2c*-het mice. *Mef2c*-het mice manifested stereotypies, including abnormal paw-clasping behavior^10,37^ (Fig. 1d) and repetitive head dipping on the hole-board exploration test^38^ (Fig. 1e). Taken together, these results suggest that *Mef2c*-het mice display a wide range of MCHS-like phenotypes and thus represent a potentially useful animal model for MCHS.

### Downregulated neurogenesis and synaptic genes in *Mef2c*-hets

**Fig. 2:**
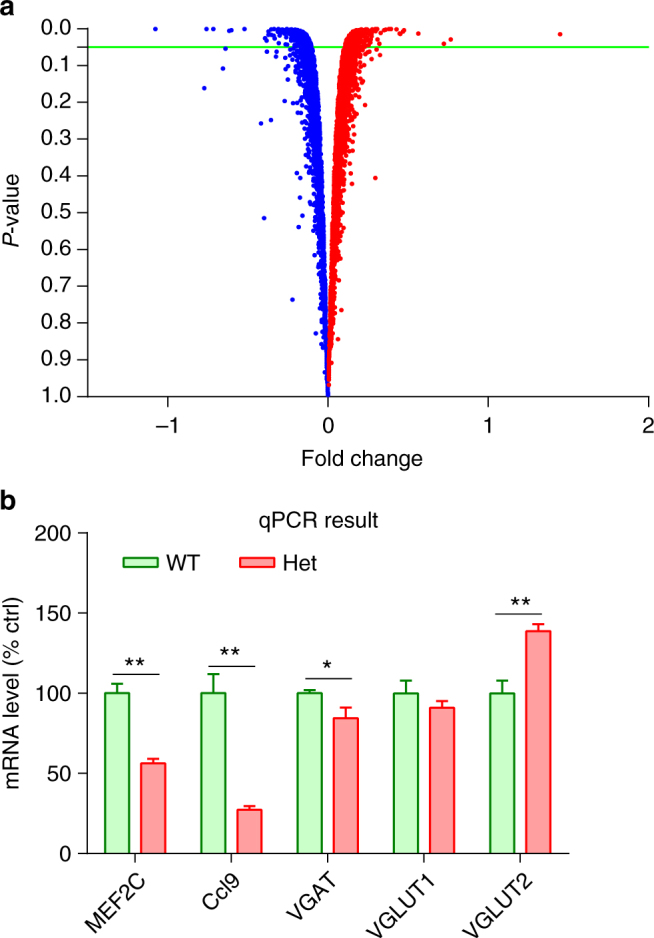
Downregulation of neurogenic and synaptic genes in *Mef2c*-het mice by microarray analysis. **a** Volcano plot shows RNA expression profiling in P30 *Mef2c*-het and WT hippocampus (red = up, blue = down, *P* < 0.05 indicated by green line). **b** Graph of qPCR experiments showing expression levels of mRNA (relative to 18S) in *Mef2c*-het mice as percentage of WT control (% ctrl; *n* = 4 per group). Data are mean ± s.e.m.; *P < 0.05, **P < 0.01 by Student’s *t* test

To identify molecular pathways underlying the pathogenesis of MCHS, we examined gene expression of *Mef2c*-het mice vs. WT littermates by microarray. We identified a total of 783 genes whose expression levels were significantly altered in the hippocampus, including 394 downregulated and 389 upregulated in *Mef2c*-het mice (Fig. 2a, above green line; Supplementary Data 1).With these data, using NextBio pathway analysis, we identified the top neuronal biogroups that were downregulated by *Mef2c* haploinsufficiency in mice, including biogroups for neurogenesis, neuronal differentiation, and synaptic function (Table 1). Concurrently, the biogroup for regulation of neuronal cell death was upregulated (Table 1). We confirmed the microarray results by quantitative PCR using RNAs extracted from 3-month-old mice (Fig. 2b). Consistent with the NextBio analysis, we found that the messenger RNA (mRNA) level of vesicular γ-aminobutyric acid (GABA) transporter VGAT (encoded by *Slc32a1*), representing an inhibitory presynaptic marker, was significantly decreased in *Mef2c*-het mice. We also examined the mRNA level of vesicular glutamate transporters 1/2 (VGLUT1/2), representing excitatory synaptic markers, and found that the level of VGLUT2, but not VGLUT1, was significantly increased in *Mef2c*-het mice. These results suggest possible dysfunction of both excitatory and inhibitory neurotransmission in these mice.

**Table 1:** Pathway analysis of all genes with altered expression in *Mef2c*-het mice

Dysregulated biogroups in *Mef2c*-het mice	# of genes dysregulated	Direction	Score	*P-*value	Source
Neurogenesis	33	Down	34.31	1.30E−15	GO
Neuron differentiation	23	Down	27.51	1.10E−12	GO
Synapse	18	Down	24.04	3.60E−11	GO
Regulation of neuron death	5	Up	9.29	9.30E−05	GO

Pathway-enrichment analysis was performed using NextBio and gene-ontology (GO-term) filtering

### Excitatory/inhibitoryneuronal deficits in *Mef2c*-hets

**Fig. 3:**
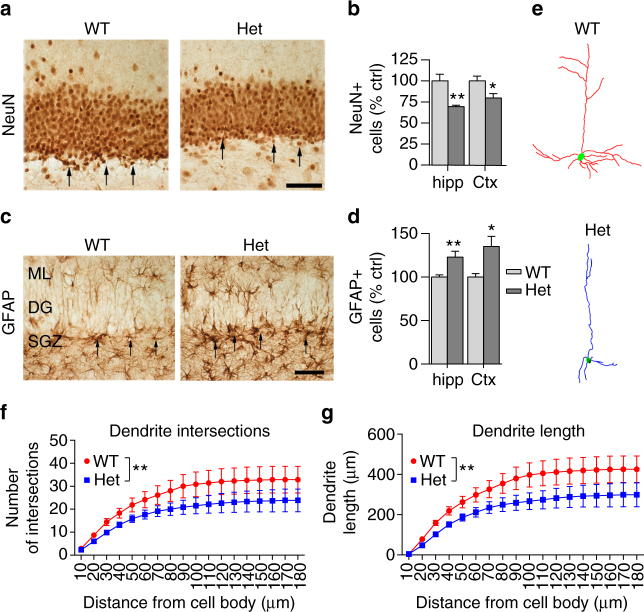
*Mef2c*-het mice exhibit abnormal neuronal properties. **a** Immunohistochemistry showing NeuN+ cells in the dentate gyrus (DG) of WT and *Mef2c*-het mice. **b** Quantification showing decreased NeuN+ cell counts in hippocampus (hipp) and cortex (Ctx) in *Mef2c*-het mice relative to WT. Hippocampal measurements were obtained on granule cells in the molecular layer of the DG, and the cortical measurement on frontal lobe layers IV and V. **c**, **d** Increased number of GFAP+ cells consistent with astrocytosis in *Mef2c*-het mice. **e** Neurolucida drawing of representative dendrites visualized by Golgi staining in V1 (primary visual cortex), M2ML (secondary visual cortex mediolateral area), and LPtA (lateral parietal association cortex) of the visual cortex of WT and *Mef2c*-het mice. **f**, **g** Summary graphs of Sholl analysis showing reduction in cumulative number of dendritic intersections (**f**) and dendritic lengths (**g**) in *Mef2c*-het neurons. Scale bar: 50 µm. Data are mean ± s.e.m.; *n* = 4 per group. *P *<* 0.05, **P *<* 0.01 by Student’s *t* test in **a**–**d** and ANOVA in **f**, **g**

In histological experiments using the optical dissector as an unbiased stereological counting method, the total number of NeuN+ cells (i.e., neurons) was significantly decreased in *Mef2c*-het mice compared to WT in the hippocampus (69.5 ± 1.6% of WT control value, *P* *<* 0.01 by Student’s *t* test) and frontal cortex (79.8 ± 5.1% of WT control, *P* *<* 0.05) (Fig. 3a, b). In contrast to NeuN+ cells, the number of glial fibrillary acid protein (GFAP)+ cells was significantly increased in *Mef2c*-het mice compared to WT in both the hippocampus (123.0 ± 6.8% of WT control, *P* *<* 0.01) and frontal cortex (135.16 ± 11.70% of WT control, *P* *<* 0.05) (Fig. 3c, d).

We next performed Golgi staining in both *Mef2c*-het and WT brains to determine dendritic branching patterns of pyramidal cells in layer V of the cerebrocortex using Neurolucida software on three-dimensional (3D) montage images (Fig. 3e). Our Sholl analyses^39^ indicated that the dendritic complexity of *Mef2c*-het neurons was significantly reduced, as demonstrated by decreased dendritic interactions (Fig. 3f) and decreased total dendritic lengths (Fig. 3g).

**Fig. 4:**
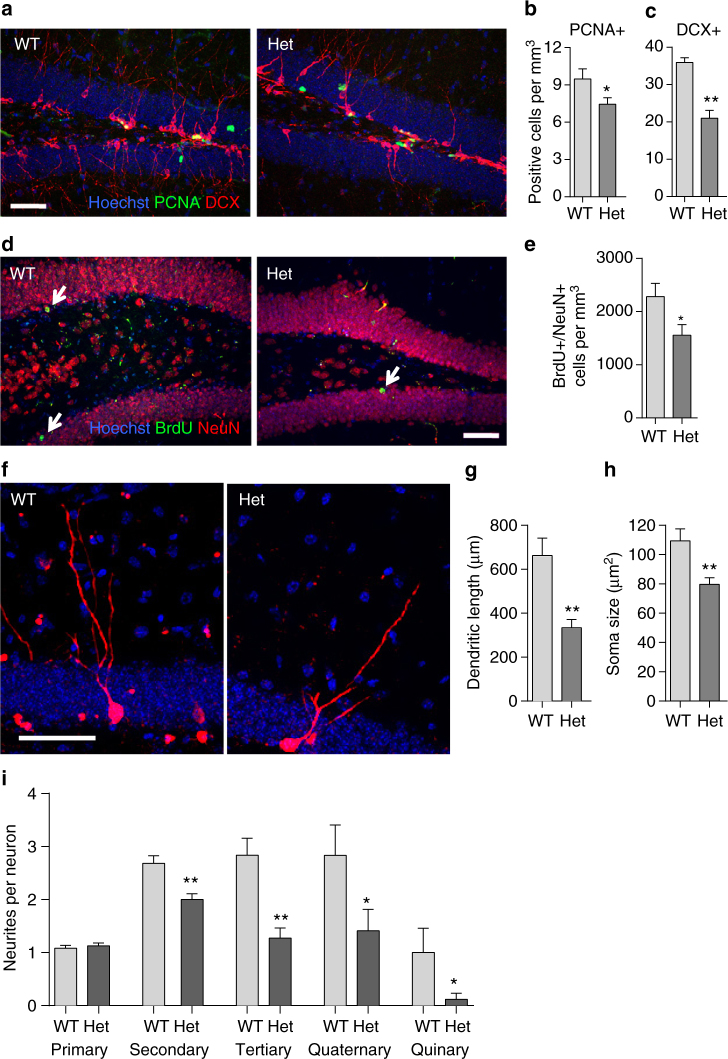
*Mef2c*-het mice exhibit abnormal adult neurogenesis. **a** Confocal images showing PCNA (green) and DCX (red) double staining in the subgranular zone (SGZ) of the DG in 8-week-old WT and *Mef2c*-het mice. **b**, **c** Quantification of PCNA+ and DCX+ cells revealed reduction in the number of proliferating cells (**b**) and developing neurons (**c**) in *Mef2c*-het DG. **d** BrdU (green) and NeuN (red) double staining 4 weeks after BrdU injection in 8-week-old WT and *Mef2c*-het mice revealed newly born DG neurons (arrows: BrdU+/NeuN+). **e** Reduction in BrdU+/NeuN+ cells in *Mef2c*-het DG; *n* = 4 mice per genotype in **a**–**e**. **f** Examples of morphological development of neurons born in adult *Mef2c*-het and WT mice. Dividing cells in the dentate gyrus (DG) were labeled with mCherry via retroviral-mediated gene transduction. Mice were killed 4 weeks later. **g** Quantification of total dendritic length of 4-week-old neurons revealed reduced dendritic length in *Mef2c-*het mice (*n* = 17) compared to WT mice (*n* = 12). **h** Quantification showed reduced somal size in *Mef2c*-het mice (*n* = 71) compared to WT mice (*n* = 28). **i** Quantification of neurite number showed normal number of primary neurites (*n*_WT_ = 25, *n*_Het_ = 55), but reduced number of secondary (*n*_WT_ = 25, *n*_Het_ = 55), tertiary (*n*_WT_ = 25, *n*_Het_ = 55), quaternary (*n*_WT_ = 12, *n*_Het_ = 17), and quinary (*n*_WT_ = 12, *n*_Het_ = 17) neurites of 4-week-old neurons in *Mef2c*-het compared to WT brain. Section thickness: 40 µm. Scale bar: 50 µm. Values are mean ± s.e.m., *P *<* 0.05; ***P *<* 0.001 by Student’s *t* test

To further account for the decrease in neuronal number, in addition to the known reduction in embryonic neurogenesis mediated by MEF2C deficiency^10^, we characterized adult neurogenesis in the subgranular zone of the dentate gyrus (DG) of 2–3 month-old *Mef2c*-het mice and found a decrease in both the number of proliferating cells (PCNA+, Fig. 4a, b) and developing neurons (DCX+, Fig. 4a, c). The number of BrdU-labeled NeuN+ cells was also reduced in the DG (Fig. 4d, e). These results suggest that reduced adult neurogenesis in *Mef2c*-het mice contributes to the reduction in neurons. In addition, the development and complexity of newly formed neurons, visualized via retroviral-mediated gene transduction of mCherry, were also decreased in the *Mef2c*-het DG, as indicated by decreased somal size and dendritic length (Fig. 4f–i). Therefore, *Mef2c* haploinsufficiency results in decreased neuronal number, impaired adult neurogenesis, and decreased dendritic complexity in mice.

**Fig. 5:**
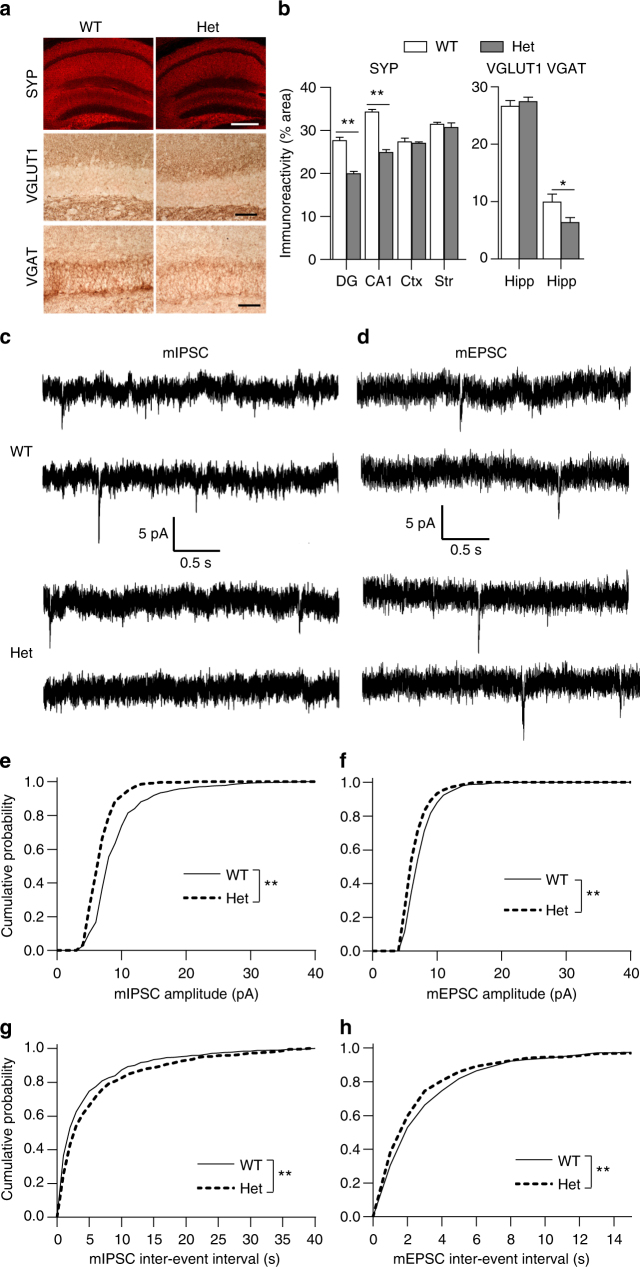
*Mef2c*-het mice exhibit altered synaptic properties and E/I imbalance in synaptic neurotransmission. **a** Immunohistochemistry of synaptophysin (SYP), VGLUT1, and VGAT in WT and *Mef2c*-het hippocampus. Scale bars: 500 µm (top panel), 50 µm (middle and bottom panels). **b** Reduced immunoreactivity of SYP in the dentate gyrus (DG) and CA1 regions of *Mef2c*-het hippocampus but not cortex (Ctx) or striatum (str, left). DG measurements were performed in the molecular layer, CA1 in the pyramidal cell layer, Ctx in frontal cortical layers IV and VI, and str in the putamen at the level of the nucleus accumbens. Reduced expression of VGAT but not VGLUT1 in *Mef2c*-het hippocampus (right). Data are mean ± s.e.m., *n* = 4 per group; *P < 0.05, **P < 0.01 by Student’s *t* test. **c**, **d** Representative traces of mIPSCs (**c**) and mEPSCs (**d**) from slice recordings of DG neurons of WT and *Mef2c*-het mice. **e**–**h** Cumulative plots of mIPSC and mEPSC amplitude and inter-event intervals. *n* = 7–9 per genotype; **P < 0.01 by two-sample Kolmogorov–Smirnov test

We subsequently examined synapses in *Mef2c*-het mice. Consistent with the microarray analysis predicting an alteration in synaptic proteins, quantitative confocal immunohistochemistry showed that expression of synaptophysin (SYP), a presynaptic marker, was significantly decreased in the hippocampus of *Mef2c*-het mice (Fig. 5a, b). To better define the synaptic deficit, we examined expression levels of the predominant excitatory synaptic protein VGLUT1 and the inhibitory synaptic protein VGAT by quantitative confocal immunohistochemistry in the hippocampus (Fig. 5a). We found that expression of VGAT, but not VGLUT1, was significantly decreased in *Mef2c*-het mice (Fig. 5b). In addition, we performed immunoblot experiments on hippocampal synaptosome-enriched lysates and found that the levels of SYP and GAD65 (another inhibitory neuronal marker), but not VGLUT1, were downregulated in *Mef2c*-het mice (Supplementary Fig. 2a). The ratio of VGLUT1 (excitatory neurons) to GAD65 (inhibitory neurons) was significantly increased in *Mef2c*-het mice (Supplementary Fig. 2b), a sign of E/I imbalance. Moreover, in contrast to VGLUT1 and in line with our mRNA findings, VGLUT2 protein, which is normally expressed only at very low levels in adult hippocampus^40,41^, was significantly upregulated in *Mef2c*-het vs. WT (Supplementary Fig. 2c). Taken together, these findings indicate aberrant excitatory and inhibitory synaptic protein expression in *Mef2c*-het hippocampus.

To determine whether these alterations in E/I marker expression are accompanied by abnormalities in functional synaptic transmission, we recorded spontaneous miniature excitatory and inhibitory post-synaptic currents (mEPSCs/mIPSCs) from hippocampal slices of *Mef2c*-het and WT mice. From the theory of quantal release, a change in miniature frequency reflects a change in presynaptic neurotransmitter release or in the number of synapses, while a change in miniature amplitude is thought to represent a change in postsynaptic function, e.g., the number of post synaptic receptors. *Mef2c*-het mice displayed decreased mIPSC frequency (manifested as increased inter-event interval in Fig. 5c, g), in line with the overall reduction in presynaptic VGAT, dendrites and synapses. Reduced mIPSC amplitude was also observed (Fig. 5c, e), possibly reflecting the fact that MEF2 levels are known to correlate with the expression of specific GABA receptor subunits^42,43^. Interestingly, these mice also showed an increase in mEPSC frequency (manifested as decreased inter-event interval, Fig. 5d, f), similar to a previous report of increased mEPSC frequency in brain-specific *Mef2c*-KO mice^7^. This result is also consistent with our finding of increased expression of presynaptic VGLUT2 in the*Mef2c*-het hippocampus. The slight reduction in mEPSC amplitude (Fig. 5d, h) may reflect the fact that MEF2 transcriptionally normally upregulates glutamate receptor expression^44^. The overall change in mIPSCs and mEPSCs would be expected to result in an elevated E/I ratio in *Mef2c*-het mice. Indeed, as determined by the quotient of mean mEPSC to mIPSC values, *Mef2c*-het mice manifested a 116.2% increase in the E/I frequency ratio and a 25.7% increase in E/I amplitude ratio compared to WT mice, confirming the existence of functional E/I imbalance.

To determine if these neuronal and synaptic defects have a deleterious effect on synaptic plasticity and neuronal circuitry, we recorded hippocampal LTP. *Mef2c*-het mice exhibited reduced LTP in the CA1 region of the hippocampus (Supplementary Fig. 3a). Paired pulse facilitation (PPF) represents short-term enhancement of presynaptic function in response to the second of two paired stimuli caused by residual Ca^2+^ in the presynaptic terminal after the first stimulation. For example, decreased PPF is associated with increased probability of neurotransmitter release. We observed a statistical decrease in PPF in *Mef2c*-het vs. WT mice (Supplementary Fig. 3b), consistent with our observed small increase in mEPSC frequency (Fig. 5d, f). Collectively, our results show that *Mef2c*-het mice manifest a reduced number of neurons, accompanied by synaptic deficits with decreased inhibitory and increased excitatory synaptic neurotransmission, thus leading to E/I imbalance.

### NitroSynapsin rescues autistic behaviors in *Mef2c*-het mice

In other mouse models of ASD, decreased GABAergic neurotransmission has been reported to contribute to E/I imbalance, leading to autistic-like social and cognitive deficits that parallel those found in humanautism^27^. Therefore, it is possible that the autistic/MCHS-like behavioral deficits in *Mef2c*-het mice may also be triggered, in part, by reduced inhibitory neurotransmission, as well as by the increased excitatory synaptic activity found here. In this regard, aminoadamantane drugs like the FDA-approved drug memantine have been reported to restore altered E/I balance^30^. Thus, we reasoned that chronic treatment with NitroSynapsin^34–36^, an aminoadamantane nitrate displaying increased efficacy at the NMDAR compared to memantine, might mitigate MCHS-like phenotypes in *Mef2c*-het mice via its ability to restore E/I balance. To test this hypothesis, we treated male *Mef2c*-het or WT mice with NitroSynapsin or PBS vehicle for 3 months. We then performed behavioral, electrophysiological, and histological analyses to determine the effects of this drug. Importantly, NitroSynapsin treatment of WT mice showed no effects on the Morris water maze, EPSCs, or LTP^36^.

**Fig. 6:**
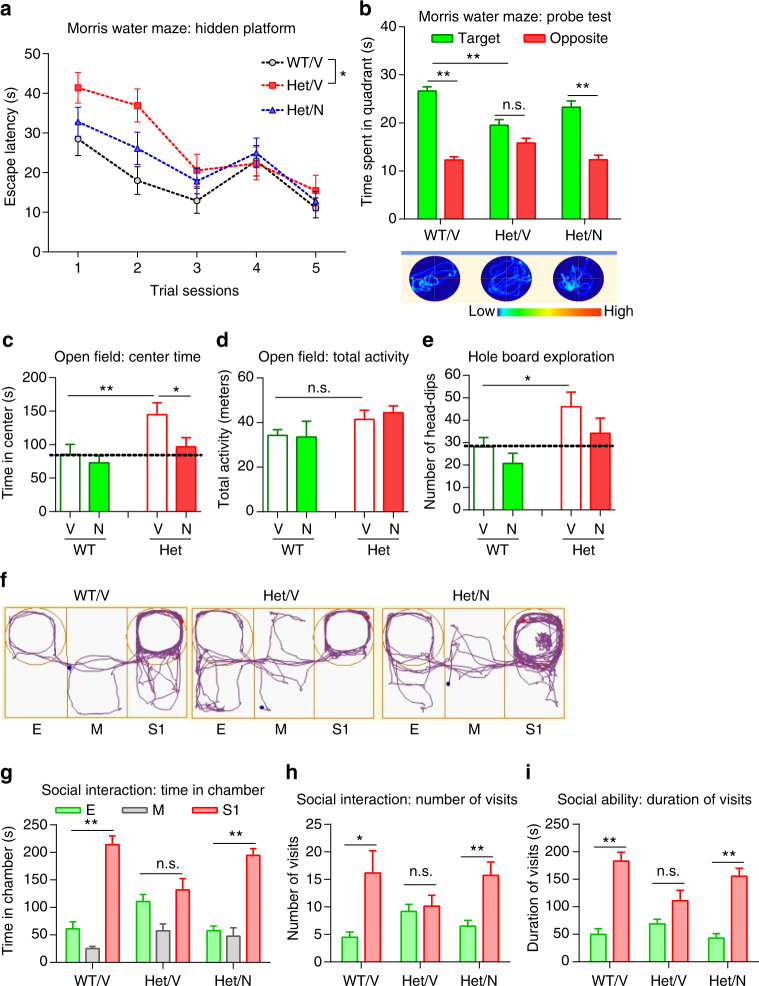
NitroSynapsin rescues MCHS-like phenotypes in *Mef2c*-het mice. **a** Latency of finding hidden platform during training sessions in the Morris water maze. **b** In the probe test, vehicle-treated *Mef2c*-het mice (Het/V) showed no preference between target and opposite quadrants, suggesting impaired memory. Treatment with NitroSynapsin (N) rescued this effect (Het/N). Representative swim patterns shown at bottom. **c**, **d** In the open field test, Het/V mice exhibited increased center time that was rescued by treatment with N (**c**). In contrast, Het/V mice displayed normal total activity (**d**). **e** Het/V mice displayed increased head-dips per hole, suggesting repetitive behavior. Treatment with N rescued. **f**–**i** N treatment rescued aberrant social ability in *Mef2c*-het mice. **f** Representative traces of mouse movement in the three-chamber social ability test. **g**–**i** *Mef2c*-het mice (Het/V) exhibit abnormalities in social interaction measured by time spent in each chamber (**g**), number of visits (**h**), and duration of visits (**i**) to E (empty) or S1 (stranger mouse 1) chambers. N treatment ameliorated this deficit (Het/N). Data are mean ± s.e.m. *n* = 7–9 per group. *P < 0.05, **P *<* 0.01 by ANOVA. M middle chamber

Neurobehavioral tests were used to determine whether treatment of *Mef2c*-het mice with NitroSynapsin could rescue autistic/MCHS-like behavioral phenotypes. We first performed the Morris water maze to test the effecton spatial learning and memory (Fig. 6a, b). During hidden platform training sessions, vehicle-treated *Mef2c*-het (Het/V) mice showed impaired spatial learning in the first 2 days by taking longer to find the hidden platform than vehicle-treated WT (WT/V) mice (Fig. 6a). However, *Mef2c*-het mice treated with NitroSynapsin (Het/N) showed improved performance relative to vehicle during these tests. This improvement cannot be attributed to an increase in swimming speed per se, neither *Mef2c* heterozygosity nor NitroSynapsin treatment affected swimming speed (Supplementary Fig. 4). Twenty-four hours after all groups of mice reached the criteria (20 s to find the hidden platform), we performed probe tests to examine memory retention. As shown in Fig. 6b, WT/V mice displayed normal memory retention by spending a significantly longer time in the target quadrant, where the hidden platform was previously located. In contrast, Het/V mice displayed impaired memory by not showing a preference to the target quadrant over the opposite quadrant. Interestingly, Het/N mice spent significantly more time in the target quadrant than in the opposite quadrant, suggesting that NitroSynapsin treatment normalized memory function (Fig. 6a, b). We next performed an open field test, a 30-min test to assay general locomotor activity. Het/V mice showed enhanced center activity (Fig. 6c), but not total activity (Fig. 6d).This abnormal behavior was rescued by chronic treatment with NitroSynapsin. The drug also corrected the abnormal repetitive behavior of increased head dipping of *Mef2c*-het mice in the hole-board exploration test (Fig. 6e). Finally, we performed a social interaction behavioral test. WT/V mice spent significantly more time in a chamber with a stranger mouse 1 (S1) than in a chamber with a similar but empty cage (E). However, Het/V mice showed no preference for time spent in either chamber (Fig. 6f, g), a sign of impaired social ability. In addition, Het/V mice paid significantly fewer visits to S1 and for shorter times per visit than WT/V mice(Fig. 6h, i). Treatment with NitroSynapsin improved this abnormal social behavior. Importantly, initial feasibility experiments, in which we had treated *Mef2c* het mice with equimolar memantine or NitroSynapsin in a head-to-head comparison, demonstrated the superiority of NitroSynapsin in these behavioral paradigms (Supplementary Fig. 5a). In addition, NitroSynapsin treatment did not significantly alter the social behavior of WT mice (Supplementary Fig. 6).

Taken together, these results show that chronic treatment of *Mef2c*-het mice with NitroSynapsin significantly improved cognitive deficits, repetitive behavior, impaired social interactions, and possibly altered anxiety. Of note, *Mef2c*-het mice did not exhibit aberrant motor behaviors except for paw clasping (Supplementary Fig. 7a–e). However, NitroSynapsin treatment did not improve the paw clasping phenotype (Supplementary Fig. 7f).

### NitroSynapsin effect on E/I neuronal markers and LTP

**Fig. 7:**
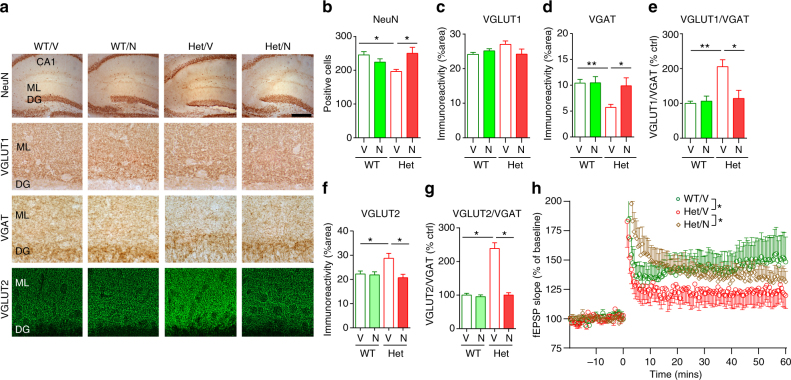
NitroSynapsin rescues abnormal neuronal and synaptic properties in *Mef2c*-het mice. **a** Immunohistochemical images of NeuN, VGLUT1, VGAT, and VGLUT2 in the molecular layer (ML) of the hippocampal dentate gyrus (DG) of WT and *Mef2c*-het mice treated with vehicle (V) or NitroSynapsin (N). Scale bars: 500 µm (top panel), 25 µm (middle panels), 40 µm (bottom panel). **b**–**f** Summary graphs showing rescue by NitroSynapsin of decreased number of total NeuN+ cell counts (**b**), reduced immunoreactivity of VGAT (**d**) and VGLUT2 (**f**), and increased ratio of VGLUT1/VGAT (**e**) or VGLUT2/VGAT (**g**) in the hippocampus of *Mef2c*-het mice. **h** Impaired LTP in *Mef2c*-het mice was also rescued by NitroSynapsin. Data are mean ± s.e.m., *n* = 4–5 per group in **a**–**g** and 7–9 in **h**. *P < 0.05, **P < 0.01, by ANOVA

We performed immunohistochemistry to determine the effects of drug treatment on neuronal loss and altered expression of VGAT or VGLUT2 in the hippocampus of *Mef2c*-het mice (Fig. 7a–g). Specifically, monitored by stereology using the optical dissector method, the total number of NeuN+ cells in the hippocampus of Het/N mice was significantly greater than in Het/V mice (Fig. 7b), consistent with the efficacy of NitroSynapsin in the prior behavioral experiments. In addition, in our initial feasibility experiments, we found a significantly greater effect of NitroSynapsin over memantine on NeuN+ cell counts (Supplementary Fig. 5b).

We found that the reduction in NeuN+ cells in *Mef2c*-het mice could be accounted for at least in part by apoptotic cell loss because the number of neurons staining for active caspase-3 and for terminal deoxynucleotidyl transferase dUTP nick end labeling (TUNEL) in the CA3 region of the hippocampus was significantly increased in *Mef2c*-het mice compared to WT (*P* < 0.012, Supplementary Fig. 8). Moreover, while the number of activated caspase 3-positive and TUNEL-positive cells was increased in Het/V, it was reduced back to normal in Het/N mice (Supplementary Fig. 8). This result is consistent with the notion that apoptotic neurons observed in *Mef2c*-het mice were significantly rescued by NitroSynapsin. Moreover, treatment with NitroSynapsin also normalized the number of GFAP+ cells with astrocytic morphology in *Mef2c*-het mice (Supplementary Fig. 9).

We next determined the effect of NitroSynapsin on altered expression of E/I markers in *Mef2c*-het mice by quantitative confocal immunohistochemistry. While the level of VGLUT1 immunoreactivity was unaltered by NitroSynapsin treatment, VGAT and VGLUT2 levels as well as the ratio of VGLUT1/VGAT or VGLUT2/VGAT were normalized by NitroSynapsin treatment in *Mef2c*-het mice (Fig. 7c–g). We also found that the numbers of both parvalbumin (PV)-expressing basket-interneurons and PV-positive synapses were significantly reduced in *Mef2c*-het mice (Supplementary Fig. 10), while NitroSynapsin significantly increased PV+ synapses (% area) (Supplementary Fig. 10). These results suggest that NitroSynapsin can restore E/I balance in *Mef2c*-het mice. Finally, chronic treatment with NitroSynapsin also significantly rescued impaired hippocampal LTP in *Mef2c*-het mice (Fig. 7h, Supplementary Fig. 11).

## Discussion

**Fig. 8:**
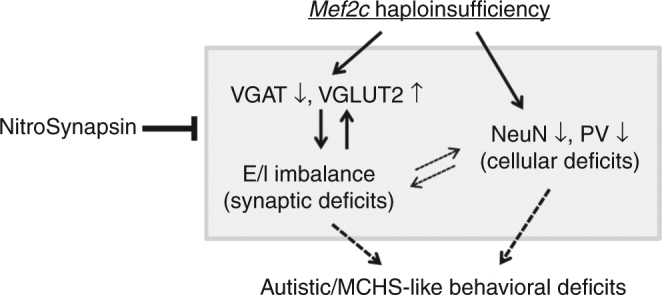
Summary diagram of *Mef2c* haploinsufficiency leading to E/I imbalance and MCHS-like phenotypes that are rescued by NitroSynapsin. *Mef2c* haploinsufficiency leads to decreased VGAT and increased VGLUT2 protein levels, resulting in E/I imbalance (overexcitability) and synaptic dysfunction. *Mef2c* haploinsufficiency also causes neuronal loss, notably a reduced number of PV+ inhibitory interneurons. These synaptic and cellular abnormalities are likely the underlying cause of the MCHS-like behavioral phenotypes observed in *Mef2c*-het mice. The histological and behavioral phenotypes are ameliorated by chronic treatment with NitroSynapsin

Genetic evidence has documented the role of *MEF2C* in multiple forms of human ASD, including MCHS^11–22^. In the current study (as summarized in Fig. 8), we provide evidence that *Mef2c*-het mice display MCHS-like behavioral deficits and thus represent a model for studying disease pathophysiology. *Mef2c*-het mice show reduced viability, the cause of which is currently unknown. Prior work has shown that systemic *Mef2c*-KO mice are embryonic lethal by E10.5 due to incomplete cardiac morphogenesis^45^; in contrast, nestin-cre-driven brain-specific *Mef2c*-KO mice exhibit reduced viability similar to that observed in the present study in *Mef2c*-hetmice^10^. It is thus possible that dysregulation of MEF2C activity in the central nervous system (CNS) is at least partially responsible for the increased lethality of *Mef2c*-het mice. The *Mef2c*-het mice that survive to adulthood exhibit a reduced number of neurons and synaptic impairment, specifically E/I imbalance caused by reduced inhibitory and enhanced excitatory neurotransmission. Importantly, treatment of *Mef2c*-het mice with the new, improved NMDAR antagonist NitroSynapsin^34–36^ not only corrects E/I imbalance, but also improves autistic/MCHS-like behavioral deficits, thus providing target validation and potential disease treatment.

We recently described the dual-functional mechanism of action of the drug NitroSynapsin^36^. Although originally termed a “NitroMemantine,” the drug is not strictly a derivative of memantine, but rather a novel aminoadamantane nitrate. The dual-functional drug acts both as an NMDAR open-channel blocker and redox modulator; in fact, the aminoadamantane moiety targets the nitro payload to the second site of action, the redox modulatory sites of the NMDAR, composed of critical regulatory cysteine residues. Recent publications have discussed the excellent CNS permeation of the drug, and its very good pharmacokinetic and phamacodynamic parameters^34–36^. Interestingly, aminoadamantane compounds like memantine have been previously shown to improve E/I imbalance^30^. In the case of NitroSynapsin, its improved action over previous aminoadamantanes at inhibiting hyperfunctioning extrasynaptic NMDARs is thought to represent the mechanism for regrowth of functional synapses that were compromised^35^, with resultant correction of E/I imbalance in the *Mef2c*-het model mice. One panoptic explanation for this effect based on our prior findings^34–36^ is that NitroSynapsin treatment, by protecting synapses, may indirectly increase excitatory input onto compromised inhibitory neurons in *Mef2c*-hets, thus enhancing their activity in order to compensate for the E/I imbalance.

Our results also show that VGAT was substantially reduced in *Mef2c*-het mouse hippocampus. In accord with this finding, functional inhibitory synaptic transmission was reduced, as demonstrated in recordings of spontaneous mIPSCs. In addition, VGLUT2 was aberrantly upregulated, consistent with an increase in excitatory neurotransmission, as documented by increased mEPSC frequency. Consequently, dysfunctional inhibitory and excitatory neurotransmission contribute to E/I imbalance in the hippocampus of *Mef2c*-het mice. This pathophysiology may contribute eventually to both synapse elimination, loss of LTP, and neuronal loss. Importantly, NitroSynapsin substantially improved all three parameters in *Mef2c*-het mice, with increases in synaptic markers, LTP, and neuronal number. Moreover, NitroSynapsin significantly improved autistic/MCHS-like behaviors in *Mef2c*-het mice. In conclusion, we demonstrate that *Mef2c*-het mice represent a useful model for human MCHS. We further show that E/I imbalance may play a role in the pathogenesis of MCHS. Restoring synaptic plasticity and preventing neuronal loss with an appropriate NMDAR antagonist can rescue or ameliorate autistic/MCHS-like phenotypes in *Mef2c*-het mice.These results may thus have implications for the treatment of human MCHS and other forms of ID and ASD.

## Methods

### Mice and drug treatments

*Mef2c* heterozygous knockout (*Mef2c*-het) mice were created on the C57BL/6J background by crossing mice carrying the conventional exon 2-deleted allele of *Mef2c* (*Mef2c* ^Δ*2*^)^45^ with their WT littermates. All procedures for maintaining and using these mice were approved by the Institutional Animal Care and Use Committee (IACUC) at the Sanford Burnham Prebys Medical Discovery Institute. In this study, only male mice were used for thebehavioral assays to insure uniformity (either with or without drug treatment). Chronic treatment with memantine^46^, NitroSynapsin (both at 4.6 µmol/kg body weight)^35,47^ or vehicle (PBS) was administered via i.p. injection, twice a day for at least 3 months, starting at ~2.5 weeks of age. This age was chosen because mice are still juveniles and thus treatment could begin in human at an equivalent stage. We chose the dose and duration of drug treatment based on previous studies in which NitroSynapsin exhibited significant protective effects on neurons and synapses^35,47^.

Mice were randomly distributed to memantine, NitroSynapsin, or vehicle groups before being genotyped. Laboratory workers performing the i.p. injections and behavioral tests were blinded to genotypes. After behavioral tests, mice were used for either immunohistochemistry or electrophysiology, as described below, and studied in a blinded fashion.

### Locomotor activity

Locomotor activity was measured in polycarbonate cages (42 × 22 × 20 cm) placed into frames (25.5 × 47 cm) mounted with two levels of photocell beams at 2 and 7 cm above the bottom of the cage (San Diego Instruments, San Diego, CA). These two sets of beams allowed recording of both horizontal (locomotion) and vertical (rearing) behavior. A thin layer of bedding material was applied to the bottom of the cage. Mice were tested for 30 or 120 min depending on the exact test.

### Paw clasping

For the paw clasping test^10,37^, mice were picked up by the distal third of their tails and observed for 10 s. They were rated in a blinded fashion with regard to genotype based on clasping of the front and/or back paws: 0—no paw clasping, 1—occasional clasping of front paws, and 3—constant clasping of front paws and occasional clasping of back paws.

### Barnes maze

The Barnes maze consisted of an opaque Plexiglas disc 75 cm in diameter, elevated 58 cm above the floor by a tripod. Twenty holes, 5 cm in diameter, were located 5 cm from the perimeter, and a black Plexiglas escape box (19 × 8 × 7 cm) was placed under one of the holes. Distinct spatial cues were located all around the maze and kept constant throughout the study. On the first day of testing, a training session was performed, which consisted of placing the mouse in the escape box and leaving it there for 1 min. One minute later, the first session was started. At the beginning of each session, the mouse was placed in the middle of the maze in a 10-cm high cylindrical black start chamber. After 10 s, the start chamber was removed, a buzzer (80 dB) and a light (400 lux) were turned on, and the mouse was set free to explore the maze. The session ended when the mouse entered the escape tunnel or after 3 min had elapsed. When the mouse entered the escape tunnel, the buzzer was turned off and the mouse allowed to remain in the dark for 1 min. When a mouse did not enter the tunnel by itself, it was gently put into the escape box for 1 min. The tunnel was always located underneath the same hole (stable within the spatial environment), which was randomly determined for each mouse. Mice were tested once a day for 12 days for the acquisition portion of the study. Note, in general, the Barnes maze is often preferred in mice over the Morris water maze because it is less stressful. However, since we had tested rodents with NitroSynapsin for other indications using the Morris water maze^36^, it was also used here for drug testing to afford comparison.

### Morris water maze

We tested spatial reference learning and memory using a version of the conventional Morris water maze^48^. The mice were trained to swim to a platform 14 cm in diameter and submerged 1.5 cm beneath the surface of the water. The platform was invisible to the mice while swimming. If a mouse failed to find the platform within 60 s, it was manually guided to the platform and allowed to remain there for 10 s. Mice were given four trials a day for as many days as necessary to reach the criterion (<20 s mean escape latency). Retention of spatial training was assessed 24 h after the last training trial. Both probe trials consisted of a 60-s free swim in the pool without the platform. The ANY-maze video tracking system (Stoelting Co.) was used to videotape all trials for automated analysis.

### Three chamber social interaction

This test was originally developed by the Crawley group^49^ for an animal model of autism. Autistic individuals show aberrant reciprocal social interaction, including low levels of social approach and unusual modes of interaction. We used a social interaction apparatus consisting of a rectangular, three chambered Plexiglas box, with each chamber measuring 20 cm (length) × 40.5 cm (width) × 22 cm (height).Walls dividing the chamber were clear with small semicircular openings (3.5 cm radius), allowing access into each chamber. The middle chamber was empty and the two outer chambers contained small, round wire cages (Galaxy Cup, Spectrum Diversified Designs, Inc., Streetsboro, OH). The mice were habituated to the entire apparatus for 5 min. To assess social interaction, mice were returned to the middle chamber, this time with a stranger mouse (C57BL/6J of the same sex tethered to the wire cage). Time spent in the chamber with the stranger mouse and time spent in the empty wire cage-containing chamber were each recorded for 5 min, as was the number of entries into each chamber. Experimental mice were tested once, and the stranger C57BL/6J mice were used for up to six tests.

### Hole board exploration

The apparatus consisted of a Plexiglas cage (32 × 32 × 30 cm) with 16 holes in a format of 4 × 4 (each 3 cm in diameter) equally spaced on an elevated floor. The explorative activity including the number of head-dips and the time spent head-dipping were measured for 5 min^50^.

### Motor behavioral tests

Balance was measured by the latency to fall off the elevated (40 cm) horizontal rod (50 cm long) in four 20 s trials. A flat wooden rod (9 mm wide) was used in trials 1–2 and a cylindrical aluminium rod (1 cm diameter) was used in trials 3–4. In each trial, the animals were placed in a marked central zone (10 cm) on the elevated rod. A score of 0 was given if the animal fell within 20 s, 1 if it stayed within the central zone for 20 s, 2 if it left the central zone, and 3 if it reached one of the ends of the bar.Traction capacity was measured over three 5-s trials as the ability of the animal to raise the hind limbs while remaining suspended by the forepaws grasped around an elevated horizontal bar (2 mm diameter). A score of 0 was given if the animal raised no limbs, 1 if it raised one limb, and 2 it raised the two limbs. Muscle Strength was determined by one trial of 60 s in which the mice were placed in the middle of the horizontal bar in an upside-down position and the latency until falling down was measured. For the vertical pole test, mice were placed with heads pointing upwards on a vertical wooden pole covered with cloth tape (1 cm diameter; height: 75 cm in trial 1, 55 cm trials 2–3). The latency to turn downward and the total time to descend to the floor over three trials was recorded. If the mouse did not turn downwards, dropped or slipped down, a default value of 60 s was recorded.

### Hippocampal slice preparation and electrophysiology

One to six-month-old mice were anesthetized with isoflurane overdose and decapitated. The brain was rapidly dissected, and hippocampal slices (350 µm in thickness) were collected in ice-cold dissection buffer containing the following (in mM): 212 sucrose, 3 KCl, 5 MgCl_2_, 0.5 CaCl_2_, 1 NaH_2_PO_4_, 26 NaHCO_3_, and 10 glucose (pH 7.4). The CA3 region was cut to avoid epileptiform activity. Slices were placed at 30 °C in artificial cerebrospinal fluid (ACSF) containing the following (in mM): 124 NaCl, 5 KCl, 26 NaHCO_3_, 1.25 NaH_2_PO_4_, 2 CaCl_2_, 1 MgCl_2_, and 10 glucose (pH 7.4). ACSF and dissection buffer were bubbled with 95% O_2_/5% CO_2_. Before recordings, slices were placed in a submersion-recording chamber, maintained at 30 °C, and perfused with ACSF for ≥1 h.

For extracellular field recordings, concentric, bipolar tungsten electrodes were used to activate Schaffer collateral/commissural (SC) fibers in the hippocampal CA1 region. Extracellular glass microelectrodes filled with ACSF (resistance ~1–3 MΩ) were placed in the stratum radiatum to measure field excitatory post-synaptic potentials (fEPSPs). For baseline recordings, slices were stimulated at 0.033 Hz for 20 min at stimulation intensities of 30–40% of those used to elicit the largest measured fEPSP amplitude. LTP was induced by applying high-frequency stimulation consisting of three 100 Hz pulses (duration: 1 s, interval: 20 s). PPF was tested by applying two pulses with interstimulus intervals ranging from 20 to 200 ms. A Multiclamp 700B amplifier (Molecular Devices) was used for experiments. Data were sampled at 5 kHz and analyzed using the Clampfit 10 program (Molecular Devices).

Synaptic activity was recorded from DG granule neurons using the whole-cell voltage-clamp technique. Data were acquired using a Multiclamp 700B amplifier and Clampex 10.2 software (Molecular Devices). Recordings were sampled at 200 µs and filtered at 2 kHz. ACSF was used as the external bath solution, with 50 µM picrotoxin and 1 µM tetrodotoxin (TTX) to isolate spontaneous mEPSCs, or 10 µM 6-cyano-7-nitroquinoxaline-2,3-dione (CNQX), 50 µM (2R)-amino-5-phosphonopentanoate(AP5), and 1 µM TTX to isolate spontaneous mIPSCs. All solutions were allowed to equilibrate for at least 20 min prior to initiating recording. The pipette internal solution for the voltage-clamp experiments contained the following (in mM): 120 K-gluconate, 15 KCl, 1 MgCl_2_, 5 HEPES, 5 EGTA, 2 Mg-ATP, pH 7.4 (300 mOsm). mEPSCs and mIPSCs were typically recorded for at least 3–5 min and analyzed using the Mini Analysis Program version 6.0.3 (Synaptosoft).

### Immunohistochemistry and unbiased stereological counting

Mice were perfused with PBS buffer and then 2% paraformaldehyde in PBS (PFA). After perfusion, brains were removed, and placed into 2% PFA overnight for post-fixation and then sunk in 30% sucrose in PBS prior to freezing. Cryostat sections were cut at a thickness of 15 µm. Sections were soaked in Antigen Unmasking Solution (Vector) and microwaved for 30 s, followed by permeabilization with 0.25% Triton X-100 in PBS for 15 min. Primary antibodies were incubated for 16 h at 4 °C and fluorescence-conjugated secondary antibodies for 2 h at 25 °C. Numerous unstained cells in each field served as an internal control for staining specificity. Primary antibodies included: NeuN (1:1000, mouse, EMD Millipore), activated caspase-3 (1:500, rabbit, Cell Signaling), VGLUT1 (1:200, guinea pig, Synaptic Systems (SYSY)), VGLUT2 (1:200, rabbit, SYSY), VGAT (1:250, mouse, SYSY), synaptophysin (1:200, mouse, Sigma), GFAP (1:500, mouse, Sigma), PCNA (1:100, mouse, Santa Cruz), and DCX (1:250, goat, Santa Cruz). TUNEL assay was performed to assess apoptosis using the Roche in situ cell death detection Kit per vendor’s instruction. The number of cells positive or percent area occupied by NeuN, activated caspase-3, TUNEL, GFAP, PCNA, or DCX was counted in specific brain regions using an optical dissector, or estimated by quantitative confocal immunohistochemistry or optical density^10,35,46^.

### Preparation of brain lysates and western blotting

Brain tissue was homogenized in 10 volumes of cold sucrose buffer (0.32 M sucrose, 25 mM HEPES, pH7.4). After a brief centrifugation at 3000×*g* for 5 min at 4 °C, the supernatant was collected and centrifuged at 10,000×*g* for 12 min at 4 °C. The outer three-fourths of the pellet was collected and re-suspended using the same sucrose buffer by gentle pipetting, while the dark center containing mitochondria was avoided. After a second centrifugation at 10,000×*g* for 12 min at 4 °C, the pellet without a dark center was collected in cold HBS (25 mM HEPES, pH 7.4, 150 mM NaCl) as the synaptosome-enriched brain lysate and used for western blot experiments^51^. Primary antibodies for immunoblotting included: VGLUT1 (1:1000, guinea pig, SYSY), VGLUT2 (1:1000, rabbit, SYSY), GAD65 (1:1000, rabbit, Millipore), synaptophysin (1:1000, mouse, Millipore), MEF2C (1:500, rabbit, Proteintech), α-tubulin (1:20,000, mouse, Sigma), and β-actin (1:10,000, mouse, Sigma) and followed by appropriate secondary antibodies^52^. Note that GAD65 was used instead of VGAT for immunoblotting because the former antibody proved superior forwestern blots. The immunosignals were captured on Kodak x-ray film and quantified using ImageJ version 1.45s (http://rsb.info.nih.gov/ij/). All uncropped western blots can be found in Supplementary Fig. 12.

### Golgi staining and Sholl analysis

Standard Golgi-Cox impregnation was performed with WT and *Mef2c*-het brains using the FD Rapid GolgiStain kit (FD NeuroTechnologies, Inc.) according to the manufacturer’s instructions. After a 3D montage of an entire cell was taken at ×40 by deconvolution microscopy and reconstructed with SlideBook 5.0 software (Intelligent Imaging Innovations), Neurolucida neuron tracing software (MBF Bioscience) was used to delineate the whole cell profile and Sholl analysis was performed, as described in detail elsewhere^39^. Cumulative dendritic intersections and dendritic lengths were analyzed.

### Adult neurogenesis

To study adult neurogenesis, 8-week-old mice were injected i.p. twice daily for 5 consecutive days with BrdU (50 mg/kg body weight) and perfused with 4% PFA 4 weeks after the last injection. Brains were then dissected and fixed overnight in 4% PFA, rinsed, cryoprotected, and frozen in liquid N_2_. Cryosections (30 or 40 µm in thickness) were sliced on a cryostat. Standard immunostaining procedures were used for primary antibodies with appropriate conjugated secondary antibodies. For BrdU immunostaining, sections were pretreated in 2 N HCL for 30 min. Cells positive for PCNA, DCX, BrdU, NeuN, or mCherry were analyzed in serial sections through the hippocampal DG of *Mef2c*-het and WT mice. We counted positive cells under a ×63 objective using SlideBook software. The total number of cells was counted using an optical dissector technique. Pictures were taken with the same exposure time and contrast/brightness parameters. The mean intensity for a particular marker was determined using ImageJ software and normalized to the average intensity of DG granule neurons. A minimum of six pictures containing at least 40 cells was analyzed for each marker.

### Microarray and NextBiogene network analysis

Total RNA was extracted from frozen tissues prepared from the hippocampi of WT and *Mef2c*-het mice at postnatal day 30, using the Qiagen miRNA kit. RNA concentrations were determined using a Nanodrop spectrophotometer (Thermo Fisher Scientific), and RNA quality was assessed using an Agilent Bioanalyzer. All RNA samples included in the expression analysis had an RNA integrity number (RIN) >8. MouseRef-8 v2 expression beadchip (Illumina) was used for the gene-expression microarray. Microarray data analysis was performed using the R software and Bioconductor packages. Raw expression data were log2 transformed and normalized by quantile normalization. Data quality control criteria included high inter-array correlation (Pearson correlation coefficients >0.85) and detection of outlier arrays based on mean inter-array correlation and hierarchical clustering.

For pathway enrichment analysis, all genes whose expression was statistically altered (*P* < 0.05) in *Mef2c*-het mice relative to WT mice were clustered for GO terms using the pathway enrichment application of NextBio (Illumina, Inc.). The background set of genes used was the entire human genome. Rank scores were assigned by NextBio^53^. Genes clustered to GO terms related to neuronal development were prioritized for validation of changes in gene expression.

### Statistical analysis

Data are reported as mean ± s.e.m. Statistical tests in each experiment are listed here, in figure legends, or in the text. All data were analyzed using the Prism 6 program **(**GraphPad Software, Inc.). For data with a normal distribution, statistical significance was determined by Student’s *t* test for pairwise comparisons. An ANOVA with Tukey’s, Dunnett’s, or Newman-Keuls post hoc analysis was used for multiple comparisons. For categorical data, a *χ* ^2^ test or Fisher’s exact test on a 2 × 2 contingency table was employed. For data not fitting a normal distribution, non-parametric tests were used. *P* < 0.05 was considered statistically significant.

### Data availability

The authors declare that all data supporting the findings of this study are available within the article and its supplementary information files or from the corresponding authors upon reasonable request. The raw data for the microarray (presented in Supplementary Data 1) have been deposited in the NCBI GEO database under accession code GSE103298.

## Electronic supplementary material

Supplementary Information

Description of Additional Supplementary Files

Supplementary Data 1
