# Supplementary material for: NitroSynapsin therapy for a mouse MEF2C haploinsufficiency model of human autism
Source: Nat Commun. 2017 Nov 14;8:1488. doi: 10.1038/s41467-017-01563-8 (PMC5684358; doi:10.1038/s41467-017-01563-8)
Supplement: Supplementary file 1 — Supplementary Information [file 41467_2017_1563_MOESM1_ESM.pdf]

Supplementary Information

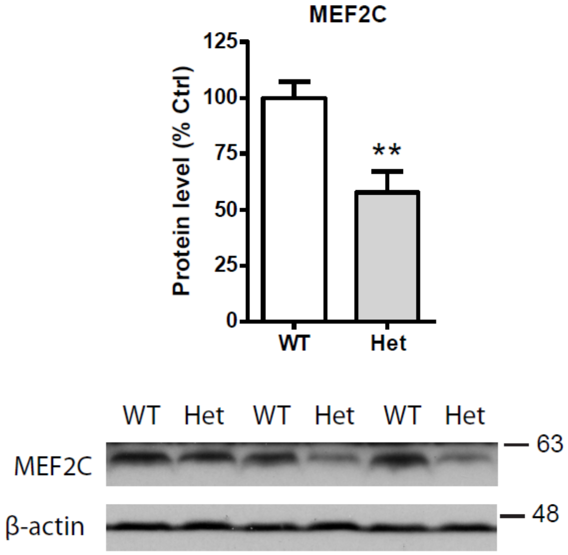

Supplementary Figure 1 | MEF2C protein levels are decreased in *Mef2c*-het brains. Immunoblotting of forebrain tissue lysates shows decreased MEF2C in *Mef2c*-het mice compared to WT. Protein levels normalized to ß-actin (% control). Representative blots illustrated at bottom. Values are mean + s.e.m., $n = 5$; $^{**}P < 0.01$ by Student's t-test.

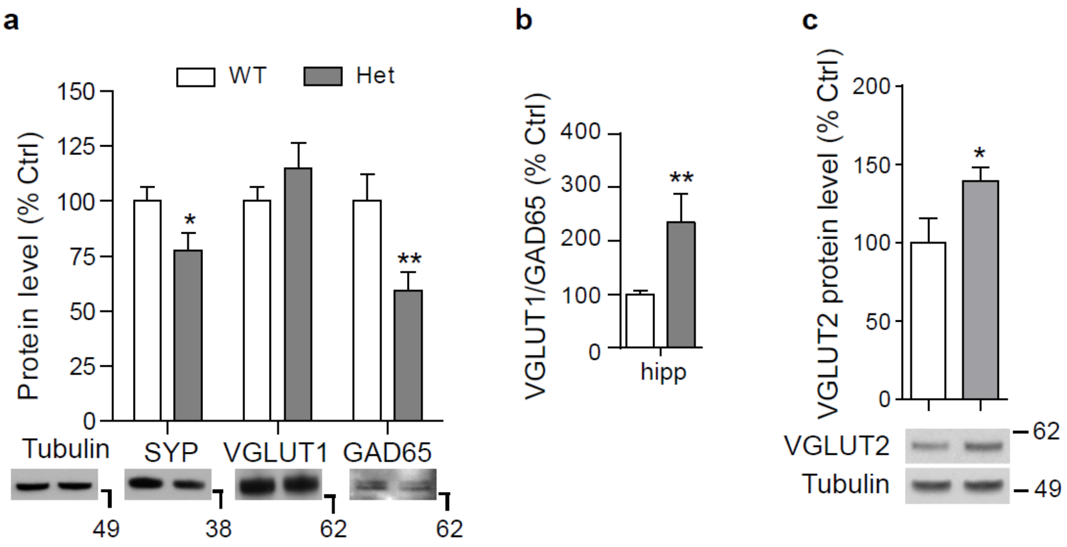

Supplementary Figure 2 | *Mef2c*-het mice express altered levels of synaptic proteins in the hippocampus. (**a**) Immunoblots of synaptosome-enriched hippocampal lysates show decreased synaptophysin (SYP) and GAD65 but not VGLUT1 in *Mef2c*-het mice. Protein levels normalized to α-tubulin (% control). Representative blots illustrated *at bottom*. (**b**) Ratio of VGLUT1 to GAD65. (**c**) Immunoblots of synaptosome-enriched hippocampal lysates show increased VGLUT2 normalized to α-tubulin in *Mef2c*-het mice. Representative blots *at bottom*. Data are mean + s.e.m., $n = 4$ per genotype; $*P < 0.05$, $**P < 0.01$. Statistical significance was determined by Student's *t*-test.

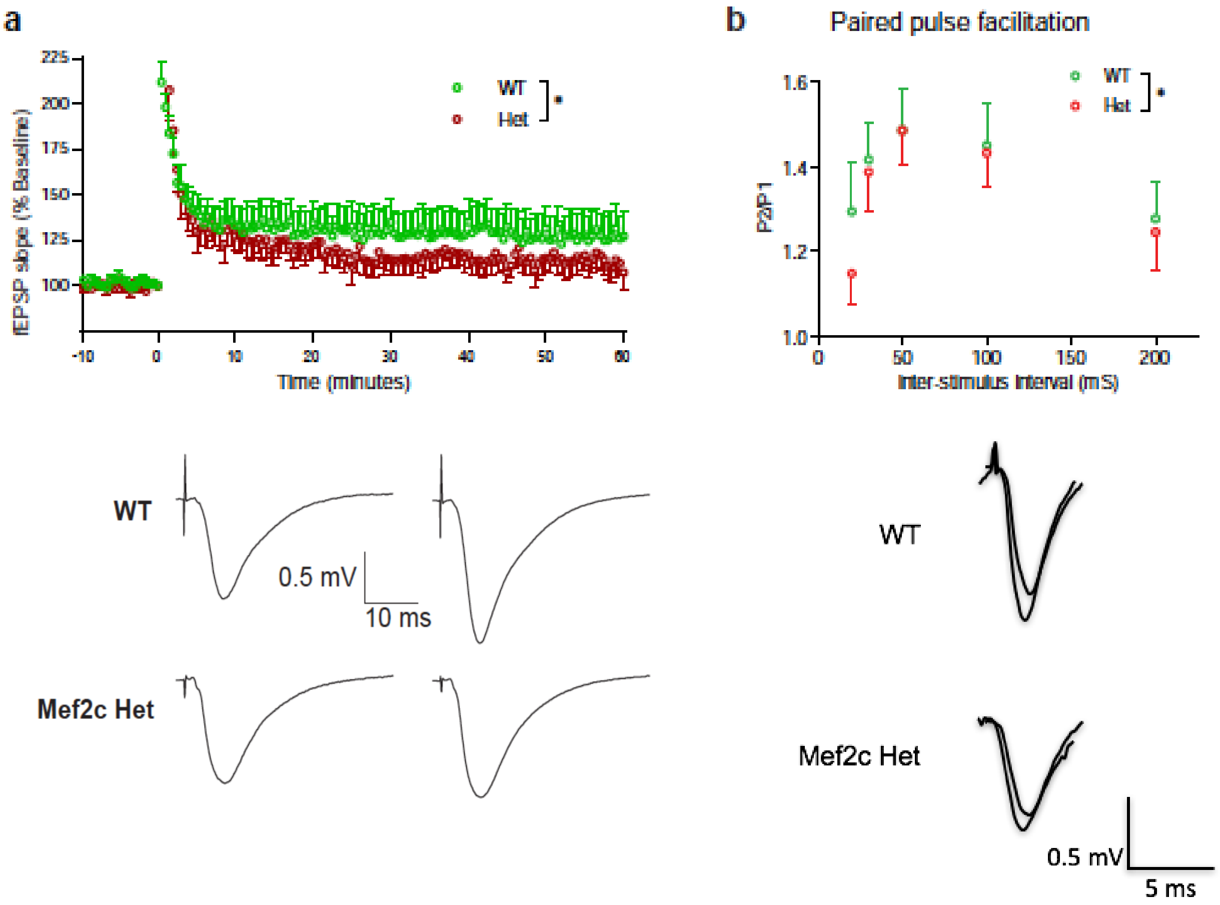

Supplementary Figure 3 | *Mef2c*-het mice exhibit impaired hippocampal LTP and PPF in slice recordings. (**a**,**b**) *Mef2c*-het mice showed impaired LTP (**a**) and decreased PPF (**b**). Representative traces are shown below the graphs. Data are mean ± s.e.m., n = 5-9 per genotype. Statistical significance was determined by ANOVA (**a**, *P < 0.01) or Sign test (**b**, *P < 0.05).

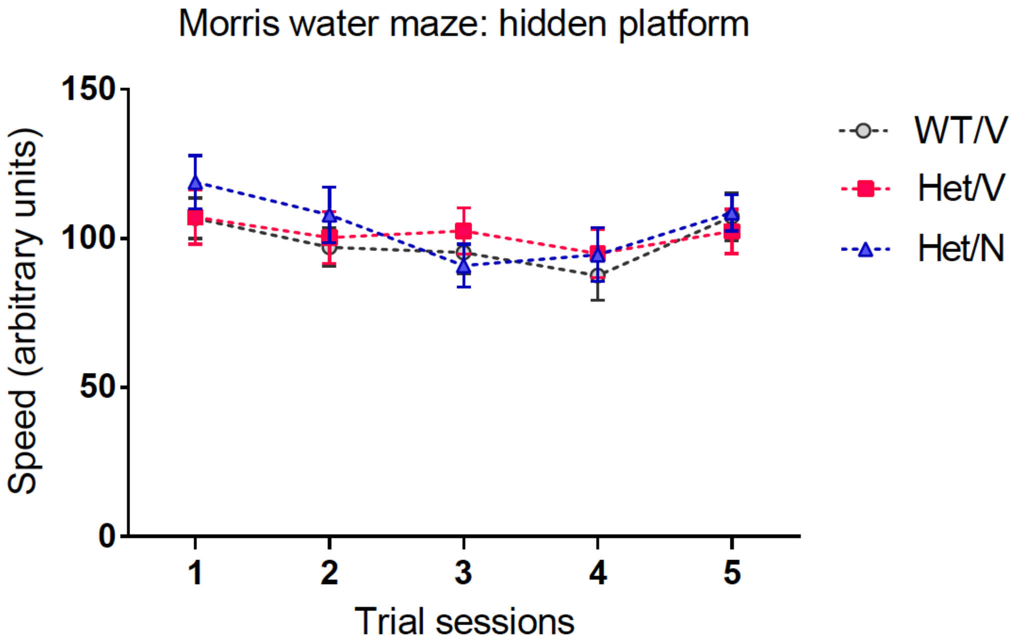

Supplementary Figure 4 | Neither *Mef2c* heterozygosity nor NitroSynapsin treatment alters swimming speed of mice in the Morris water maze. The speed of mice escaping to the hidden platform during the training phase of the Morris water maze was calculated as distance divided by latency. Unlike the latency to the platform (see **Fig. 6**), the speed of the three groups of mice (WT/V, Het/V, and Het/N) was not significantly different. Data are mean ± s.e.m, n = 7-9 per group.

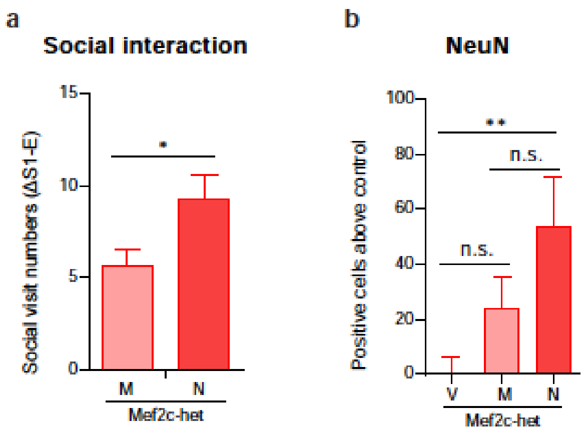

Supplementary Figure 5 | NitroSynapsin is more effective than memantine in rescuing neurological deficits of *Mef2c*-het mice. (**a**) Summary graph showing that the number of social visits by *Mef2c*-het mice treated with NitroSynapsin (N) was significantly greater than that of memantine (M)-treated mice in the three-chamber social interaction test. Social visits constitute the number of visits to the chamber with a stranger mouse (S1) minus the number of visits to the empty chamber (E). Data are mean + s.e.m., n = 7-9 per group; *$P < 0.05$ by Student's t-test. (**b**) Summary graph showing that the number of hippocampal NeuN+ cells in *Mef2c*-het mice treated with NitroSynapsin (N), but not memantine (M), was significantly greater than in vehicle (V)-treated mice. Data are mean + s.e.m., n = 4-5 per group; **$P < 0.01$ by ANOVA.

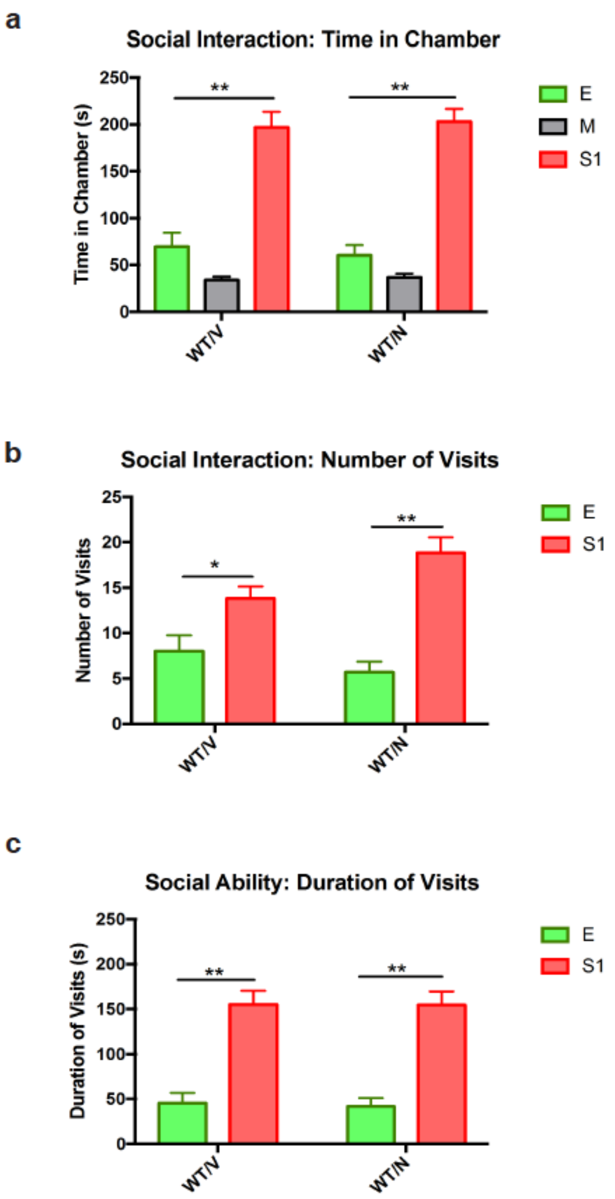

Supplementary Figure 6 | NitroSynapsin (N) treatment does not alter the social behavior of WT mice in the three-chamber assay. Summary graphs showing time in three chambers (**a)**, number of visits (**b**), and duration of visits (**c**) by WT mice after 3-month treatment with vehicle (V) or NitroSynapsin (N). NitroSynapsin treatment did not significantly alter the social behavior of WT mice. Data are mean + s.e.m., n = 10 each for WT/V and WT/N groups. E: Empty chamber; S1: Stranger mouse 1 chamber. *P < 0.05 and **P < 0.01, compared to E by ANOVA or t-test.

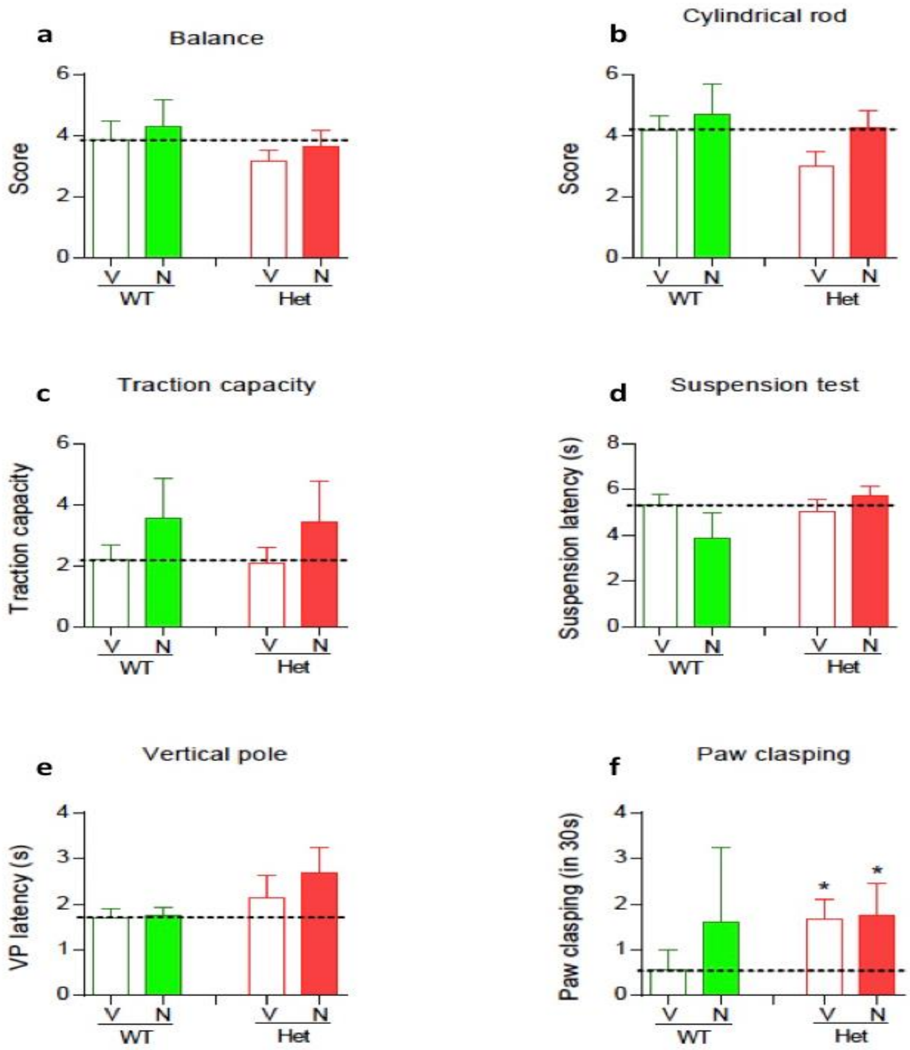

Supplementary Figure 7 | *Mef2c*-het mice do not exhibit abnormal motor behaviors except paw clasping which is not rescued by NitroSynapsin. Summary graphs showing motor behaviors of WT and *Mef2c*-het mice after 3-month treatment with vehicle (V) or NitroSynapsin (N). *Mef2c*-het mice did not exhibit significantly altered behaviors on the balance beam (**a**), cylindrical rod (**b**), traction capacity (**c**), suspension test (**d**), or vertical pole tests (**e**). In these experiments, treatment with NitroSynapsin had no significant effect on behaviors of either WT or *Mef2c*-het mice. The abnormal paw clasping activity observed in *Mef2c*-het mice was not rescued by NitroSynapsin treatment (**f**). Data are mean + s.e.m., n = 12, 5, 11, and 13 for WT/V, WT/N, Het/V, and Het/N groups, respectively. *$P < 0.05$ compared to WT/V by ANOVA.

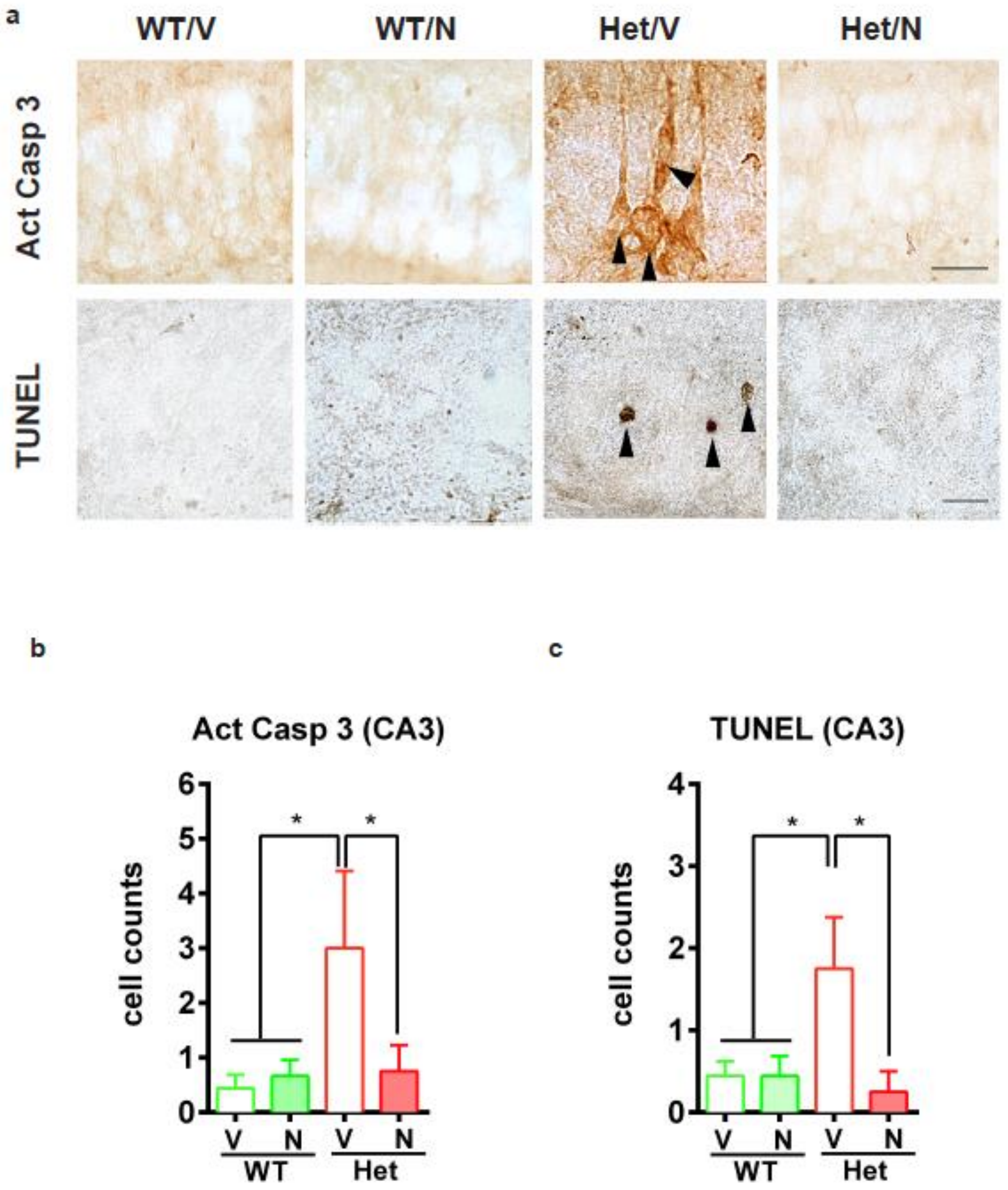

Supplementary Figure 8 | *Mef2c*-het mice show increased caspase-3 activation and apoptosis in the hippocampus. (**a**) Immunohistochemistry of activated caspase-3 (Act Casp 3, *top panels*) and TUNEL staining (*bottom panels*) in the hippocampal CA3 region of WT mice treated with vehicle (WT/V) or NitroSynapsin (WT/N), and in *Mef2c*-het mice treated with vehicle (Het/V) or NitroSynapsin (Het/N). Scale bars, 25 µm**.** (**b,c**) Histogram showing that caspase-3+ (**b**) and TUNEL+ neurons (**c**) were significantly increased in Het/V mice compared to control WT/V mice. Furthermore, both of these phenotypes were ameliorated in Het/N mice. Data are mean + s.e.m., n = 4-5 per group; *$P < 0.05$, by ANOVA.

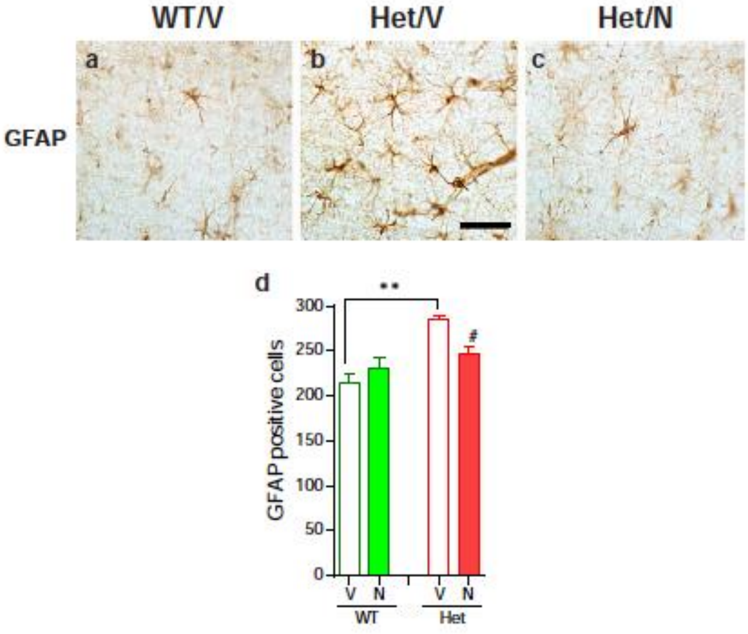

Supplementary Figure 9 | NitroSynapsin normalizes the number of astrocytes in *Mef2c*-het hippocampus. (**a-c**) Representative images showing increased GFAP+ cells with morphology of astrocytes in *Mef2c*-het mice vs. WT (**b** vs. **a**). The number of GFAP+ cells was restored to WT levels by chronic treatment with NitroSynapsin (**c**). Images from molecular layer of the hippocampal dentate gyrus. Scale bar, 25 µm. (**d**) Quantification of GFAP+ cells in the hippocampus of WT and *Mef2c*-het mice treated with vehicle (V) or NitroSynapsin (N). Data are mean + s.e.m., $n = 4$ or 5 per group; $^{**}P < 0.01$ compared to WT/V and $^{\#}P < 0.05$ compared to Het/V by ANOVA.

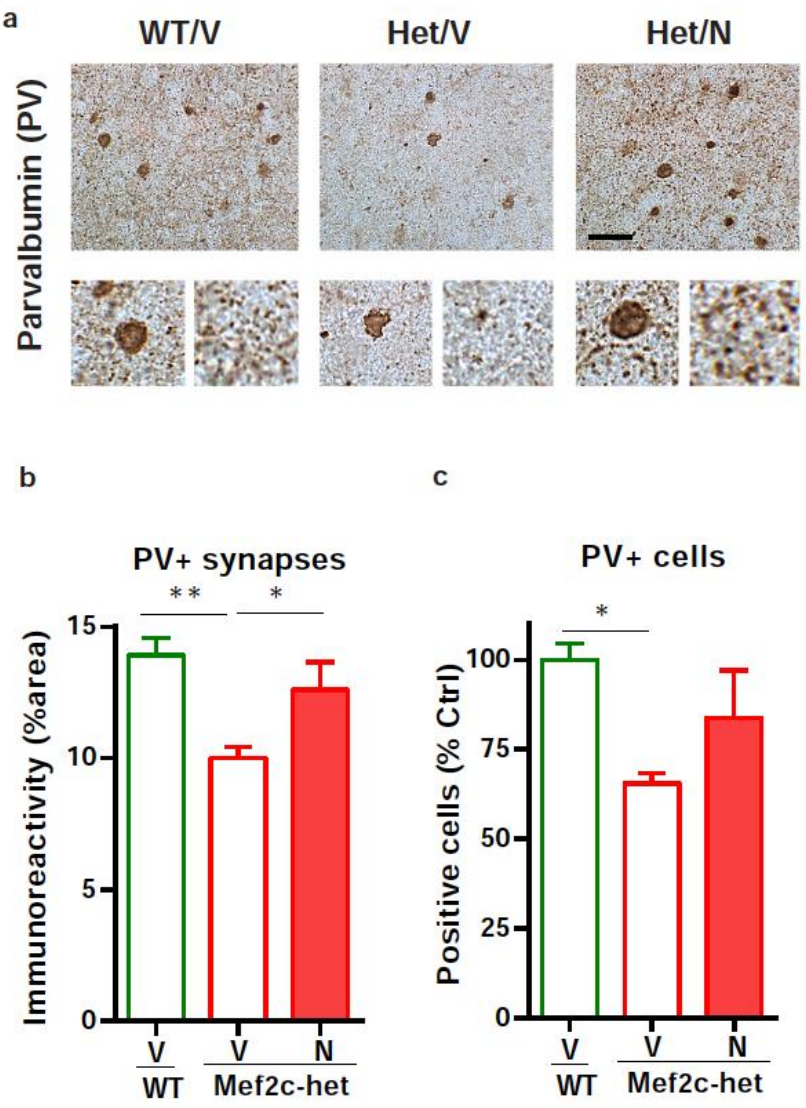

Supplementary Figure 10 | *Mef2c*-het mice display reduction in parvalbumin+ (PV+) inhibitory synapses and cells. (**a**) Representative images showing PV+ immunoreactivity in the hippocampus of WT mice treated with vehicle (WT/V) and in *Mef2c*-het mice treated with vehicle (Het/V) or NitroSynapsin (Het/N). Higher magnification of PV+ neurons (*bottom left of each panel*) or synapses (*bottom right of each panel*). Scale bar, 25 µm. (**b**) Histogram showing reduction in PV-immunoreactive synapses in Het/V mice compared to WT/V mice. This reduction was significantly ameliorated after NitroSynapsin treatment. (**c**) Histogram showing reduction in the number of PV-immunoreactive cells in Het/V mice but not in Het/N mice compared to WT/V mice. Data are mean + s.e.m., n = 4-5 per group; *P < 0.05, **P < 0.01 by ANOVA.

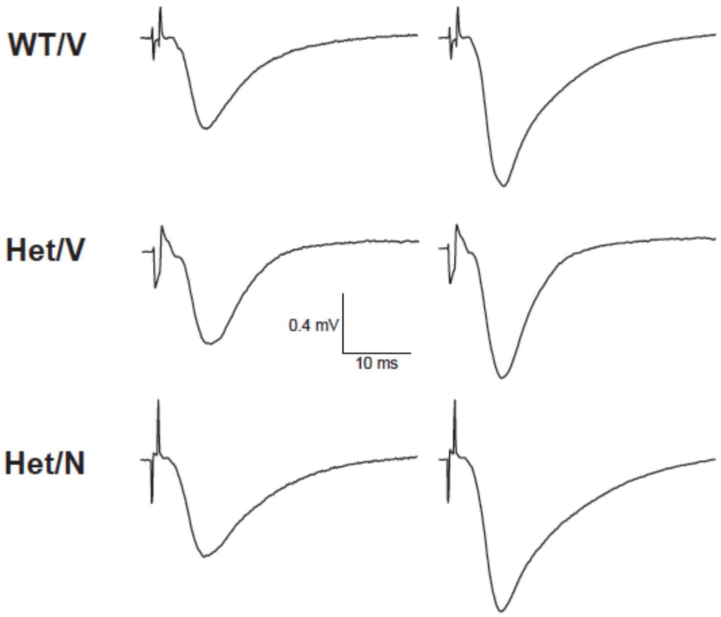

Supplementary Figure 11 | NitroSynapsin treatment rescues deficits in LTP in *Mef2c*-het mice. Representative traces of evoked currents before (*left*) and after (*right*) induction of hippocampal LTP in slices prepared from WT/V-, Het/V-, and Het/N-treated mice. fEPSP slopes for each group of animals is presented in **Fig. 7h** in the text.

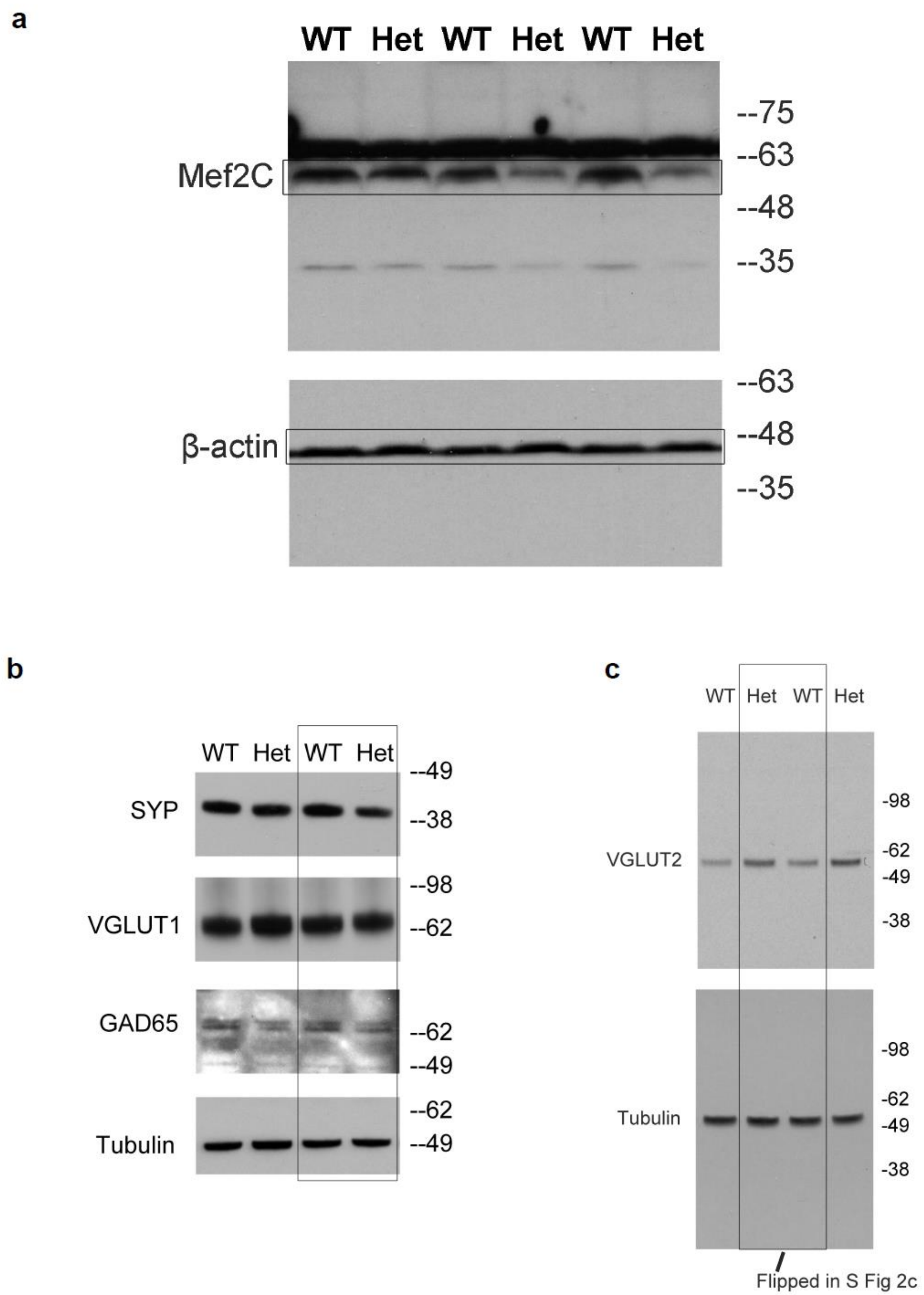

Supplementary Figure 12 | Uncropped scans of blots. (**a**) Supplementary Figure 1. (**b**) Supplementary Figure 2a. (**c**) Supplementary Figure 2c.
