# Supplementary material for: NitroSynapsin therapy for a mouse MEF2C haploinsufficiency model of human autism
Source: Nat Commun. 2017 Nov 14;8:1488. doi: 10.1038/s41467-017-01563-8 (PMC5684358; doi:10.1038/s41467-017-01563-8)
Supplement: Supplementary file 2 — Description of Additional Supplementary Files [file 41467_2017_1563_MOESM2_ESM.docx]

**Description of Additional Supplementary Files**

File Name: Supplementary Data 1

Description: Results for differential gene expression (sorted by decreasing *P*-values) between WT and *Mef2c*-het for hippocampus at postnatal day 30 (*n* = 4 per group) as analyzed using limma package in R. Shown are the log2 Fold change (log2FC), t-statistics (t), *P*-value (*P*.Value), FDR corrected P-value (adj.P.Val), b-statistics (B), Z-scores in *Mef2c*-het samples (compared to WT). Normalized expression values for each sample are provided.
